# Inferring the functions of longevity genes with modular subnetwork biomarkers of *Caenorhabditis elegans *aging

**DOI:** 10.1186/gb-2010-11-2-r13

**Published:** 2010-02-03

**Authors:** Kristen Fortney, Max Kotlyar, Igor Jurisica

**Affiliations:** 1 Department of Medical Biophysics, University of Toronto, 610 University Avenue, Toronto, M5G 2M9, Canada; 2 The Campbell Family Institute for Cancer Research and Ontario Cancer Institute, 101 College Street, TMDT 9-305, Toronto, M5G 1L7, Canada; 3 Department of Computer Science, University of Toronto, 10 King's College Road, Toronto, M5S 3G4, Canada

## Abstract

A central goal of biogerontology is to identify robust gene-expression biomarkers of aging. Here we develop a method where the biomarkers are networks of genes selected based on age-dependent activity and a graph-theoretic property called modularity. Tested on *Caenorhabditis elegans*, our algorithm yields better biomarkers than previous methods - they are more conserved across studies and better predictors of age. We apply these modular biomarkers to assign novel aging-related functions to poorly characterized longevity genes.

## Abstract 2

An algorithm for determining networks from gene expression data enables the identification of genes potentially linked to aging in worms.

## Background

Aging is a highly complex biological process involving an elaborate series of transcriptional changes. These changes can vary substantially in different species, in different individuals of the same species, and even in different cells of the same individual [1-3]. Because of this complexity, transcriptional signatures of aging are often subtle, making microarray data difficult to interpret - more so than for many diseases [4,5]. Interaction networks represent prior biological knowledge about gene connectivity that can be exploited to help interpret complex phenotypes like aging [6,7]. Here for the first time, we integrate networks with gene expression data to identify modular subnetwork biomarkers of chronological age.

With few exceptions, previous analyses of aging microarray data have been limited to studying the differential expression of individual genes. However, single-gene analyses have been criticized for several reasons. Briefly, they are insensitive to multivariate effects and often lead to poor reproducibility across studies [8-10] - even random subsets of data from the same experiment can produce widely divergent lists of significant genes. Recent studies have shown that examining gene expression data at a systems level - in terms of appropriately chosen groups of genes, rather than single genes - offers several advantages. Compared to significant genes, significant gene groups are more replicable across different studies, lead to higher performance in classification tasks, and are more biologically interpretable [8,11].

Many complementary approaches to the systems-level analysis of microarray data have been proposed. These range from methods like Gene Set Enrichment Analysis [12], which determines whether members of pre-defined groups of biologically related genes (such as those supplied by the Gene Ontology (GO) [13]) share significantly coordinated patterns of expression, to machine learning methods that consider all possible combinations of genes and identify groups whose combined expression pattern can distinguish between different phenotypes - with no constraint that the genes in a group must be biologically related.

Network methods for interpreting gene expression data [11,14-19] fall in between these two extremes: they incorporate prior biological knowledge in the form of an interaction network - so that genes in a significant group are likely to participate in shared functions - but they consider many different combinations of genes, and so are more flexible than methods using pre-defined gene groups. Gene groups identified by these methods constitute novel biological hypotheses about which genes participate together in common functions related to the class variable.

Here, we propose a novel strategy for identifying subnetwork biomarkers: we incorporate a measure of topological modularity into the expression for subnetwork score. This yields subnetwork biomarkers that are biologically cohesive and that have different activity levels at different ages. Using two aging microarray datasets, we show that our method improves on previous approaches, yielding subnetworks that are more conserved across studies, and that perform better in a machine learning task. We identify the subnetworks that play a role in worm aging, and then explore their connection with known longevity genes. Finally, we apply them to assign putative aging-related functions to longevity genes (genes that affect lifespan when deleted or perturbed). Worm is the ideal model organism for studying these questions, since it has the largest number of characterized longevity genes [20], and microarray datasets using worms of four or more ages are publicly available [2,21]. Our work builds on a family of successful algorithms that incorporate supervised information to find subnetworks with phenotype-dependent activity, which we discuss below.

### Methods for extracting active subnetworks by integrating gene expression data, network connectivity, and supervised class labels

To date, some of the most successful network-based methods of gene group identification for class prediction have been the score-based subnetwork markers originally proposed in Ideker *et al. *[22] and developed and expanded in later works, for example, [11,14,15,18,23,24]. Subnetworks identified using these approaches were recently shown to be highly conserved across studies and to perform better than individual genes or pre-defined gene groups at predicting breast cancer metastasis [11].

Most of these methods share the same basic architecture. Each algorithm aggregates genes around a seed node in a way that maximizes some measure of performance. In previous implementations, the score is a function of the subnetwork activity (often calculated as the mean expression value of the genes in the subnetwork) and the class label - that is, subnetworks get high scores if their activity is different for different classes. Subnetworks are grown outward iteratively from a seed node, typically using a greedy search procedure to maximize subnetwork score: at every step, the network neighbor of the current subnetwork yielding the largest score increase is added to the subnetwork.

Subnetwork scores are calculated differently in individual implementations (for example, [18] uses the t-statistic and [11] uses mutual information) but are always solely a function of what we refer to as class relevance, that is, of expression data and class labels. In particular, in all previous implementations the subnetwork score is insensitive to network topology - the only topological constraint is that subnetwork members must form a connected component.

However, a large body of work in network theory has demonstrated the value of more sophisticated topological measures of network cohesiveness, or modularity [25,26]. In fact, many algorithms successfully identify groups of functionally related genes on the basis of network topology alone. The simple intuition behind these algorithms is that genes that are members of a highly interconnected group (that is, only sparsely connected to the rest of the network) are more likely to participate in the same biological function or process. In biological networks, genes belonging to the same topological module are more likely to share functional annotations or belong to the same protein complex [27-29].

No score-based subnetwork method proposed to date takes advantage of the rich modular structure of biological interaction networks. Here, we propose incorporating topological modularity into the expression for subnetwork score, and show that this approach offers important advantages - increased conservation across studies, and improved performance on a learning task. For the remainder of the paper, we refer to subnetworks grown using scores that are a function of class relevance alone as regular subnetworks, and to those grown using our new scoring criterion as modular subnetworks.

## Results and discussion

### Identifying active subnetworks in aging by trading off network modularity and class relevance

**Figure 1:**
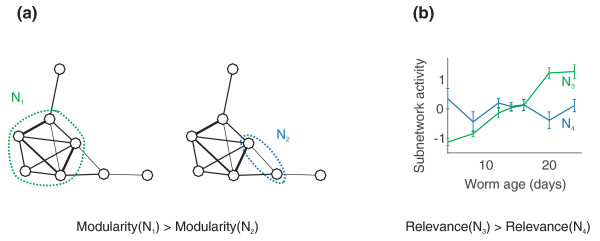
**High-scoring subnetworks fulfill two criteria: they are modular and related to aging**. **(a)** High-scoring subnetworks have high modularity, that is, they are highly interconnected, and sparsely connected to the rest of the network. **(b)** High-scoring subnetworks have high class relevance, that is, they have activity levels that increase or decrease as a function of worm age.

Here, we give a basic outline of our method for identifying subnetworks that are both highly modular and relevant to the class variable (Figure 1), and then we discuss the novel aspect - the subnetwork scoring method - in detail; other algorithm parameters are listed in Materials and methods. We compared the performance characteristics of modular and regular subnetworks using two microarray studies of worm aging [2,21].

#### Identifying modular subnetworks

**Figure 2:**
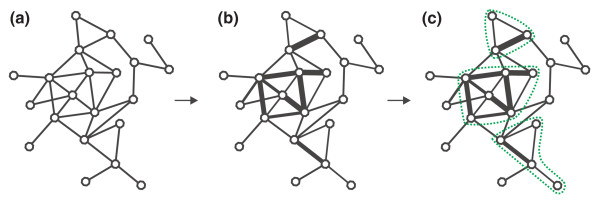
**Identifying modular subnetworks**. **(a) **Start with the largest connected component of the functional interaction network representing all genes whose expression has been measured. **(b) **Weight every edge of the network with the absolute value of the Spearman correlation between the two genes flanking it. **(c) **Identify age-related subnetworks by growing subnetworks iteratively out from seed nodes.

Our method is summarized in Figure 2. First, we assign a weight to every edge in the interaction network that reflects the strength of the relation between the two genes that flank it (quantified using Spearman correlation). For genes *i *and *j *with normalized expression vectors **z**_*i *_and **z**_*j*_, the weight *w*_*ij *_is defined as:

Next, we grow subnetworks starting at particular seed genes in the network (see Materials and methods). At each stage of the network growth procedure, the algorithm considers all network neighbors of the current subnetwork *N*. For each neighbor, the algorithm calculates the change in subnetwork score that would result if that neighbor were added to *N*. Here, we define the subnetwork score *S *as a weighted sum of class relevance *R *and modularity *M*, where *R *captures how related subnetwork activity is to age and *M *measures subnetwork cohesiveness:

At every stage, the neighbor that leads to the highest score increase (without reducing either class relevance or modularity) is added to the subnetwork.

The intuition behind the modularity parameter *M *is that it allows us to trade off the information in gene expression data with the prior knowledge about gene connectivity encoded in the functional interaction network: for noisy microarray studies, or ones with few samples, we should place a greater emphasis on prior knowledge by choosing higher values for *β*. Previous subnetwork scoring algorithms effectively assume that *β *= 0, or *S = R*.

#### Class relevance *R*

We measure class relevance as the Spearman correlation between subnetwork activity and age, so that a subnetwork is considered age-related to the extent that its activity level either increases or decreases monotonically with increasing age (Figure 1b). Subnetwork activity is calculated as the mean expression level of subnetwork genes. Thus, if the genes in subnetwork *N *have normalized expression vectors {**z**_1_, ..., **z**_*n*_}, and **c **is the vector of ages for each sample, then the activity is , and the class relevance is *R *= |*corr*(**a**, **c**)|.

#### Network modularity *M*

To define the modularity of a connected set of genes in a network, we use a weighted generalization of the local measure proposed in Lancichinetti and Fortunato [30]. We calculate the modularity for a subnetwork as the edge weight internal to the subnetwork divided by the total edge weight of all subnetwork nodes, squared. For subnetwork *N*, we define the internal, external, and total weight:

Then the modularity of *N *can be written as . For all subnetworks, *M *lies between 0 and 1.

#### Comparing regular and modular subnetworks

To compare the performance of regular and modular subnetworks, we generated several subnetworks of each type by adjusting algorithm parameters. For modular subnetworks, we set the modularity coefficient *β *= 50, 100, 250, 500, or 1,000 (significant subnetworks generated using these parameters are called m1, m2, m3, m4 and m5). For regular networks we set *β *= 0, and halted subnetwork growth at different score cutoff thresholds r = 0.01, 0.02, 0.05, 0.1 or 0.2 (groups of significant subnetworks are called r1, r2, r3, r4, and r5).

We generated modular subnetworks m1 to m5 and regular subnetworks r1 to r5 separately for two different *C. elegans *aging microarray datasets: 104 microarrays of individual wild-type (N2) worms over 7 ages (9 to 17 microarrays per age) [2], and 16 microarrays of pooled sterile (fer-15) worms over 4 ages (4 microarrays per age) [21]. For each study, we grew subnetworks seeded at every node in the functional interaction network, so that corresponding subnetworks grown using different expression datasets could be directly compared. We used randomization tests to determine which subnetworks were significantly associated with age in each study. For further details, see Materials and methods. Below, we compare these regular and modular subnetworks in terms of their robustness across studies and performance on a machine learning task.

### Modular subnetworks are more robust across studies than regular subnetworks

**Figure 3:**
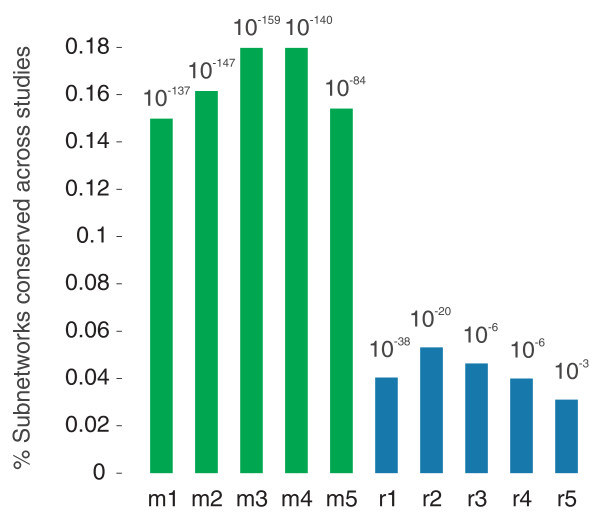
**Modular subnetworks are highly conserved across studies**. Modular subnetworks m1 to m5 are shown in green and regular subnetworks r1 to r5 in blue. Bar height shows the percentage overlap across studies for seed genes of significant modular and regular subnetworks derived from the data in Golden *et al. *[2] and Budovskaya *et al. *[21]; this is calculated as the size of the intersection of sets of significant seed genes from both studies, divided by the union. *P*-values above each bar show the significance of the overlap calculated using the hypergeometric test.

Comparing the modular subnetworks m1 to m5 and the regular subnetworks r1 to r5 derived from both studies, we found that modular subnetworks identified as significant in one study were highly likely to be significant in the other study (that is, seed genes of significant modular subnetworks were highly conserved across studies). Figure 3 shows that 15 to 18% of significant modular subnetworks were identified in both studies; in contrast, only 3 to 5% of significant regular ones were.

For each modular and regular network type, we also calculated the significance of the overlap between sets of significant seed genes using the hypergeometric test, and these values showed the same trend (Figure 3). While all subnetwork types were more conserved across studies than would be expected by chance (*P *< 10^-3^), modular subnetworks were much more conserved than regular ones - they had enrichment *P*-values ranging from 10^-84 ^to 10^-137^, while regular subnetworks had *P*-values from 10^-3 ^to 10^-38^.

While substantially more modular than regular subnetworks were conserved across studies, many subnetworks were identified in only one study; this can be partially accounted for by noise in the individual microarray studies, the fact that the two studies used different microarray platforms and different strains of worm, and the fact that the current functional interaction network is not complete and contains some errors.

### Modular subnetworks trained on aging gene expression data from wild-type worms successfully predict age in fer-15 worms

**Figure 4:**
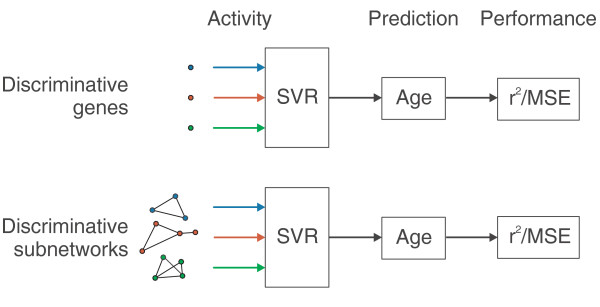
**Predicting worm age using machine learning**. The activities of genes or subnetworks (subnetwork activity is calculated as the mean activity of its member genes) are used by support vector regression (SVR) algorithms to predict age on the basis of gene expression. Performance is typically measured using both the mean-squared error (MSE) of the difference between true and predicted ages, and the squared correlation coefficient between true and predicted ages.

We compared the performance of single genes, regular subnetworks, and modular subnetworks on a machine learning task: predicting worm age on the basis of gene expression levels (Figure 4). We acquired sets of significant genes from [2]; g1 is made up of all the genes considered significant in that study, and g2 is the aging gene signature used for machine learning in [2] (that is, g2 is the 100 most significant genes from g1). Using machine learning features drawn from gene sets g1 to g2, regular subnetworks r1 to r5, or modular subnetworks m1 to m5 derived from the larger microarray study [2], we trained support vector regression (SVR) algorithms to predict the age of wild-type worms on the basis of gene expression (for details, see Materials and methods). We then tested the performance of the learned feature weights on an independent data set in a different strain of worm (fer-15) [21]. Performance on the test set was quantified as the squared correlation coefficient (SCC) between worm ages predicted by the SVR and true worm ages (measuring performance in terms of mean-squared error would be inappropriate here, because the worms in the training and test sets had different lifespans). All *P*-values reported in this section were calculated using the Wilcoxon rank-sum comparison of medians test.

**Figure 5:**
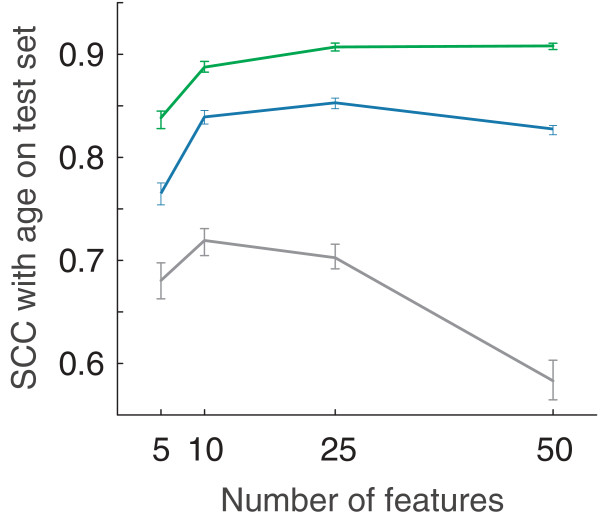
**Subnetworks and genes predict the age of fer-15 worms**. Modular subnetworks are shown in green, regular subnetworks in blue, and gene sets in gray. This figure shows the best-performing type of modular subnetworks, regular subnetworks, and genes at each feature level. For modular subnetworks, this is type m3 at every feature level; for regular subnetworks, type r3 at 5 and 10 features, r2 at 25 features, and r4 at 50 features; for genes, g2 at all feature levels. Support vector regression algorithms using 5, 10, 25, or 50 features were trained to predict age on the data from Golden *et al. *[2] and tested on Budovskaya *et al. *[21]. For each size of feature set, 1,000 different support vector regression learners were computed; curves show their median performance (quantified using the squared correlation coefficient (SCC) between true and predicted age in the bottom panel), and error bars indicate the 95% confidence intervals for the medians (calculated using a bootstrap estimate).

To capture the typical performance of machine learners that used either genes or subnetworks as features, we considered four different sizes of feature set (5, 10, 25, or 50 features). Then, for each size of feature set, and for each set of genes (g1 to g2) or subnetworks (r1 to r5, m1 to m5), we performed 1,000 tests. For example, for the 25-feature SVRs, and for the m1 significant subnetworks, we randomly drew 25 subnetworks from m1, trained them on the wild-type worm data, and then tested them on the fer-15 data - and repeated that process of drawing, training, and testing 1,000 times. Figure 5 summarizes test results at each feature level, showing the typical performance of the best sets of genes, regular subnetworks, and modular subnetworks. Full results for every parameter setting are available in Additional file 1, and *P*-value comparisons in Additional file 2.

Over all tests, the SVRs using 25 or 50 modular subnetwork features (of the m1 and m3 types) achieved the highest typical performance, with a median SCC of 0.91 between predicted and true worm age; this is a statistically significant 7% and 26% improvement over the best performances of regular subnetworks (*P *< 10^-83^) and genes (*P *< 10^-202^), respectively (Figure 5).

#### Subnetworks versus genes

Modular and regular subnetworks dramatically outperform significant genes across a range of parameters. For example, using 25 features (Figure 5), the best modular subnetworks have a median SCC of 0.91 and the best regular subnetworks of 0.85, versus 0.70 for the 100-gene signature. This result was consistent across feature levels and parameter settings, and is highly significant for all tests: that is, for every comparison between modular subnetwork features and gene features, we have *P *< 10^-15^. For all sizes of feature set, the best-performing subnetworks (m3) always showed a median SCC at least 0.16 higher than the best-performing genes (g2), that is, at least a 24% improvement.

#### Modular versus regular subnetworks

For all sizes of feature set, the median SCC of the best modular subnetwork type always exceeded that of the best regular subnetwork type by 0.05 to 0.08, corresponding to a 6 to 10% performance improvement (Figure 5). The performance difference between the best modular subnetworks and the best regular subnetworks is highly significant at all feature levels (*P *< 10^-32^).

It was not only the best modular subnetworks that outperformed the best regular subnetworks; in fact, modular subnetworks significantly outperformed the best regular subnetworks for most parameter settings. With the exception of m5 (*β *= 1,000), each modular subnetwork type significantly outperforms the best regular subnetwork type at all feature levels. For three types of modular subnetwork (m1 to m3), the performance difference between them and the best regular subnetworks is highly significant (rank-sum *P *< 10^-26 ^for every comparison); m4 outperforms the best regular subnetworks at *P *< 10^-5 ^for three feature levels, and at *P *< 10^-2 ^for five features; for m5, there is no consistent trend (Additional file 1). All pairwise comparisons (*P*-values) between regular and modular subnetworks are available in Additional file 2.

#### The role of the modularity coefficient *β* in machine learning

Different values of *β *correspond to giving different proportional weights to the information in gene expression data and to the prior knowledge about gene connectivity encoded in the functional interaction network: for noisy microarray studies, or ones with few samples, we might want to depend more on prior knowledge by choosing a high value for *β*.

For the Golden *et al. *dataset [2] that we used for training, we found that a value of *β *= 100 corresponds roughly to treating class relevance and modularity as equally important in the expression for subnetwork score: in simulations where we generated subnetworks using either modularity or class relevance alone as the scoring criterion (that is, *S = M *or *S = R*), the median modularity of the *S = M *subnetworks was two orders of magnitude smaller than the median class relevance of the *S = R *ones, that is, 'good' values for modularity are roughly 100 times smaller than 'good' values for class relevance.

As *β *becomes larger, the proportional contribution of class relevance to the expression for subnetwork score becomes smaller - and so for large enough values of *β*, the algorithm will behave essentially like other purely unsupervised network clustering algorithms that greedily aggregate nodes around a seed to maximize modularity [29-31]. In our tests, subnetworks generated using *β *= 50, 100, or 250 behaved virtually identically on the learning task; the performance of *β *= 500 subnetworks was typically a bit lower; and that of *β *= 1,000 ones lower still. For large enough values of *β*, we would expect the typical performance of modular subnetworks to fall below that of regular subnetworks, because supervised feature selection is superior to unsupervised feature selection [32].

In the previous two sections, we established that modular subnetworks are more robust across studies than regular subnetworks and perform better in a worm age prediction task. Modular subnetworks grown using the coefficient *β *= 250 showed both the highest robustness across studies and the best performance on the test set, so we chose to analyze them in greater detail. For the remainder of the paper, we will explore the relation between these subnetwork biomarkers (generated from the larger microarray study [2]) and worm aging. The full set of these subnetworks is available in Additional file 2.

### Modular subnetworks predict wild-type worm age with low mean-squared error

Here, we show using 5-fold cross-validation that modular subnetworks grown using *β *= 250 can predict the age of individual wild-type worms in the original dataset (104 worm microarrays over 7 ages) with low mean-squared error and a high SCC. Again, we used support regression algorithms (SVRs) for all learning tasks.

Because it would be circular to predict age on the same dataset that was used to determine the features [33], we first divided the wild-type worm aging dataset into five stratified folds for cross-validation. We repeated the search for significant subnetworks five times, each time using four-fifths of the data to select significant subnetworks and train SVRs, and then the remaining fifth as a test set to evaluate the learned feature weights. We compared the performance of modular subnetworks with that of the top 100 differentially expressed genes reported in [2]. To construct SVRs using genes as features, we used the same five stratified folds - that is, we used four-fifths of the data to select the top 100 most significant genes and learn feature weights, and the remaining fifth as test data, and repeated this process for each of the five folds. As in the original study [2], for each fold we selected the top 100 significant genes by performing an F-test and applying a false discovery rate [34] (FDR) correction.

For four different sizes of feature set (5, 10, 25 or 50), we generated 1,000 different SVRs using either modular subnetworks or genes as features to capture their typical performance. All *P*-values reported here were computed using the Wilcoxon rank-sum test.

**Figure 6:**
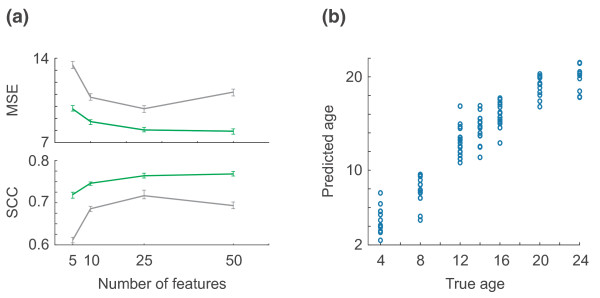
**Modular subnetwork biomarkers of aging predict the age of individual wild-type worms**. **(a) **Machine learners built from modular subnetworks or genes, predicting worm age in a cross-validation task on the data from Golden *et al. *[2] using 5, 10, 25, or 50 features. For each size of feature set, 1,000 different support vector regression learners were computed; curves show their median performance (quantified using mean-squared error (MSE) in the top panel, and the squared correlation coefficient (SCC) between true and predicted age in the bottom panel), and error bars indicate the 95% confidence intervals for the medians (calculated using a bootstrap estimate). **(b) **The performance of a typical support vector regression learner built using 50 modular subnetworks as features; true worm age is shown on the x-axis, and predicted age on the y-axis.

At every size of feature set (5, 10, 25 or 50), modular subnetworks significantly outperform differentially expressed genes (*P *< 10^-28^) according to the metrics of mean-squared error (MSE) and SCC between predicted age and true age. For example, using feature sets of size 50, we obtained a median MSE of 7.9 for subnetworks versus 11.2 for genes (*P *< 10^-98^), and a median SCC of 0.77 for subnetworks versus 0.69 for genes (*P *< 10^-65^). Figure 6a shows the median performance of modular subnetworks and genes across all tests, and Figure 6b shows the predictions of a typical SVR learner built using 50 modular subnetworks as features. At every size of feature set, the MSE for genes was at least 1.76 higher than the corresponding MSE for subnetworks (that is, at least 22% higher than the corresponding MSE for subnetworks) (*P *< 10^-28^), and the SCC for subnetworks was at least 0.05 higher (*P *< 10^-28^).

Over all tests, the modular SVRs with 50 features achieved the best performance: a median SCC of 0.77 and a median MSE of 7.9. This SCC is substantially lower than the highest one achieved on the test set of pooled fer-15 worms in the last section (0.91) because predicting the age of an individual worm is more difficult than predicting the age of a large pooled group of age-matched worms (pooling removes individual variability).

### Longevity genes play crucial roles in significant subnetworks

For these analyses, we compiled two sets of known longevity genes (see Materials and methods; Additional file 3): L1, a set of 233 genes that extend lifespan when perturbed, and L2, a larger set of 494 genes that either shorten or extend lifespan when perturbed.

#### Significant subnetworks are enriched for known longevity genes

We found that significant subnetworks derived using both *C. elegans *aging microarray studies [2,21] were significantly enriched for both sets of longevity genes, relative to the background set of 12,808 genes represented in the functional interaction network. All *P*-values reported here were calculated using the hypergeometric test. For the Golden *et al. *data [2], of the 1,957 genes that play a role in significant subnetworks, 65 are in L1 (*P *< 10^-6^) and 124 are in L2 (*P *< 10^-8^), and of the 535 seed genes that produce significant subnetworks, 27 are in L1 (*P *< 10^-5^) and 45 are in L2 (*P *< 10^-6^). For the Budovskaya *et al. *study [21], subnetwork seeds were highly enriched for known longevity genes, and the set of all subnetwork genes was slightly enriched for them. Of the 1,559 seed genes of significant subnetworks, 43 are in L1 (*P *= 0.003) and 90 are in L2 (*P *< 10^-4^), and of the 4,158 genes represented in some subnetwork, 88 are in L1 (*P *= 0.048) and 181 are in L2 (*P *= 0.025).

#### Examples of significant subnetworks containing known longevity genes

While high-throughput experimental methods have helped to identify hundreds of worm longevity genes [20], their aging-related functions remain poorly understood. We found that subnetwork biomarkers are highly enriched for longevity genes. Thus, subnetworks can provide a molecular context for these genes in aging: they can be applied to uncover new connections between different longevity genes, or to assign putative aging-related functions to them.

**Figure 7:**
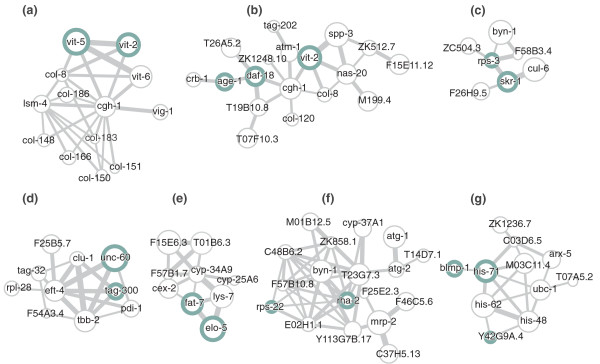
**Some examples of significant longevity subnetworks**. **(a-g) **Examples of significant modular subnetworks from Golden *et al. *[2] containing multiple known longevity genes (from L2; see Materials and methods). Edge width is proportional to gene-gene co-expression, node size is proportional to the Spearman correlation between gene expression and age, and known longevity genes are indicated by green circles.

In Figure 7, we show several representative examples of significant subnetworks derived from the Golden *et al. *data [2] that involve multiple known longevity genes. The complete list is given in Additional file 3; individual NAViGaTOR XML [35] and PSI-MI XML [36] files for each subnetwork are available from the supplementary website [37]. Subnetwork **A **involves longevity genes vit-2 and vit-5. **B **has known longevity genes age-1, daf-18, and vit-2; previous work has uncovered that a mutation in daf-18 will suppress the lifespan-extending effect of an age-1 mutation [38]. **C **contains longevity genes rps-3 and skr-1, which are involved in protein anabolic and catabolic processes, respectively. Subnetwork **D **contains longevity genes unc-60 and tag-300, which are both involved in locomotion. **E **contains longevity genes fat-7 and elo-5, which are involved in fatty acid desaturation and elongation. Subnetwork **F **has longevity genes rps-22 and rha-2, and **G **has longevity genes blmp-1, his-71, and Y42G9A.4. Blmp-1 and his-71 are both involved in DNA binding.

### Modular subnetworks participate in many different age-related biological processes

Aging is highly stochastic and affects many distinct biochemical pathways. We analyzed the union of all genes in significant modular subnetworks using biological process categories from the GO [13] and pathways from the Kyoto Encyclopedia of Genes and Genomes (KEGG) [39] databases to determine their relation to known mechanisms of aging. Full results are given in Tables 1 and 2; all functions and pathways shown in the table and discussed below are significant at *P *< 0.05 after an FDR correction.

**Table 1:** Gene Ontology biological process categories enriched in the set of genes represented in modular subnetworks

Gene Ontology biological process	*P*-value
*Translation*	6.45E-17
Hermaphrodite genitalia development	1.20E-16
Embryonic cleavage	1.37E-15
Germline cell cycle switching, mitotic to meiotic cell cycle	8.32E-14
*Locomotory behavior*	1.84E-13
*Meiosis*	1.10E-11
Positive regulation of multicellular organism growth	4.25E-11
Morphogenesis of an epithelium	3.85E-06
Protein catabolic process	1.13E-05
Phosphate transport	4.99E-04
Negative regulation of multicellular organism growth	8.07E-04
*Ubiquitin-dependent protein catabolic process*	1.94E-03
Nucleosome assembly	1.97E-03
Establishment of nucleus localization	2.37E-03
*Tricarboxylic acid cycle*	3.26E-03
DNA replication	4.64E-03
*Protein transport*	5.01E-03
*Energy coupled proton transport, against electrochemical gradient*	5.02E-03
Leucyl-tRNA aminoacylation	5.02E-03
Collagen and cuticulin-based cuticle development	5.12E-03
Organelle organization and biogenesis	5.19E-03
Chromosome segregation	7.48E-03
mRNA metabolic process	8.44E-03
Protein import into nucleus	1.15E-02
Purine base biosynthetic process	1.15E-02
Sulfur compound biosynthetic process	1.40E-02
DNA repair	1.45E-02
*Determination of adult life span*	1.74E-02
Threonine metabolic process	1.75E-02
Water-soluble vitamin biosynthetic process	1.78E-02
*ATP synthesis coupled proton transport*	3.14E-02
rRNA processing	3.85E-02
Isoleucyl-tRNA aminoacylation	4.02E-02
*Methionyl-tRNA aminoacylation*	4.02E-02
*Valyl-tRNA aminoacylation*	4.02E-02
*Embryonic pattern specification*	4.04E-02
Regulation of cell cycle	4.04E-02

All categories shown are significant at *P *< 0.05 after an FDR correction for multiple testing. GO categories written in italics are also enriched for known longevity genes (Additional file 4).

**Table 2:** KEGG pathways enriched in the set of genes represented in modular subnetworks

KEGG pathway	*P*-value
*Ribosome*	2.17E-27
*Metabolic pathways*	2.70E-15
Proteasome	2.33E-10
Pyrimidine metabolism	1.34E-09
Purine metabolism	7.08E-07
DNA replication	1.54E-06
Nucleotide excision repair	1.81E-05
*Aminoacyl-tRNA biosynthesis*	2.80E-05
*Cell cycle*	4.37E-05
Glutamate metabolism	1.54E-04
*Glycolysis/gluconeogenesis*	2.97E-04
*Citrate cycle (TCA cycle)*	5.41E-04
*Methionine metabolism*	1.25E-03
Ubiquitin mediated proteolysis	7.19E-03
Pyruvate metabolism	7.27E-03
Base excision repair	7.38E-03
*Glyoxylate and dicarboxylate metabolism*	7.39E-03
Arginine and proline metabolism	8.35E-03
Glycine, serine and threonine metabolism	8.38E-03
*Pentose phosphate pathway*	1.23E-02
Valine, leucine and isoleucine biosynthesis	1.30E-02
One carbon pool by folate	1.30E-02
RNA polymerase	1.76E-02
Alanine and aspartate metabolism	1.76E-02
Non-homologous end-joining	2.15E-02
*Selenoamino acid metabolism*	2.17E-02
Mismatch repair	2.20E-02

All categories shown are significant at *P *< 0.05 after an FDR correction for multiple testing. KEGG pathways written in italics are also enriched for known longevity genes (Additional file 4).

In total, we identified 27 KEGG pathways and 37 non-redundant GO biological processes (see Materials and methods) that were significantly enriched for subnetwork genes. To test whether these pathways and processes were also related to aging, we calculated the significance of their overlap with the set of experimentally determined longevity genes (Additional file 4). We found that one-third of the GO biological processes (12 of 37) and KEGG pathways (10 of 27) associated with subnetworks were significantly enriched for longevity genes (*P *< 0.05). Aging-associated GO categories enriched for subnetwork genes include 'locomotory behavior,' which has recently been proposed as a biomarker of physiological aging [2], and 'determination of adult life span'; KEGG pathways include 'cell cycle' and several metabolic pathways (including 'citrate cycle,' 'glycolysis').

### Modular subnetworks can be used to annotate longevity genes with novel functions

An important advantage of subnetwork over single-gene biomarkers is that they can be applied to infer novel functions for subnetwork members [40]. Most worm longevity genes were identified in high-throughput RNA interference screens, and thus many remain poorly characterized. And though several longevity genes do have some previously known functions, their aging-related function is still unknown.

We used modular subnetworks (derived from the expression data in [2]) to assign putative functions in aging to known longevity genes by annotating them with the GO biological process categories that their associated subnetworks were significantly enriched for. In total, we provided 49 longevity genes with novel annotations; 9 of these genes had no previous GO biological process annotations (apart from those electronically inferred) or well-characterized orthologs (named NCBI KOGs [41]). The most significant novel annotation for each longevity gene is given in Table 3, as an example of our approach (poorly characterized genes are indicated with an asterisk). The full list of all longevity gene GO categories inferred by subnetwork annotations is available in Additional file 5, and on the supplementary website [37]. All GO categories in the tables are significant with *P *< 0.05 (after an FDR correction), and annotated to at least 25% of subnetwork genes.

**Table 3:** Assigning putative functions to longevity genes

Gene	Gene Ontology biological process	*P*-value
*rpl-4*	Cellular macromolecular complex assembly	2.16E-02
*vit-5*	Phosphate transport	3.70E-05
*rha-2*	Cellular macromolecular complex assembly	2.16E-02
*C06E7.1*	Protein complex assembly	2.26E-02
C25H3.6*	Transcription from RNA polymerase II promoter	4.87E-02
*pat-4*	Chromatin assembly or disassembly	4.92E-03
*C33H5.18*	Chromatin assembly or disassembly	3.02E-03
*unc-60*	Protein complex assembly	2.26E-02
*vit-2*	Phosphate transport	3.70E-05
ril-1*	Cell adhesion	3.57E-02
CD4.4*	Ribosome biogenesis	1.85E-02
*eif-3.F*	Organelle organization and biogenesis	3.75E-03
F09F7.5*	Pigment metabolic process	5.01E-03
*pab-2*	Chromatin assembly or disassembly	8.99E-05
*hpk-1*	Growth	2.78E-02
*mdh-1*	Lipid metabolic process	3.36E-02
*blmp-1*	Chromatin assembly	7.22E-04
*daf-3*	Protein complex assembly	2.26E-02
F28B3.5*	Amine metabolic process	3.04E-03
*rps-23*	tRNA aminoacylation for protein translation	1.04E-03
*F30A10.10*	Chromatin assembly or disassembly	4.95E-02
*dlk-1*	Transcription from RNA polymerase II promoter	4.87E-02
F40F8.5*	Nucleobase metabolic process	5.08E-05
*elo-5*	Lipid metabolic process	4.34E-02
*F43G9.3*	Water-soluble vitamin metabolic process	2.04E-03
*ife-1*	Organelle organization and biogenesis	3.75E-03
*spt-4*	Chromatin assembly or disassembly	8.40E-05
*aakb-1*	Nucleobase, nucleoside and nucleotide metabolic process	1.45E-03
dod-22*	Gene expression	1.85E-02
*F57B9.3*	Amine metabolic process	2.83E-02
*cdc-25.1*	Amine metabolic process	1.90E-02
*nac-3*	Cellular macromolecular complex assembly	2.16E-02
*lin-23*	Cytoskeleton organization and biogenesis	2.59E-02
*K10D2.2*	Anion transport	5.54E-04
*ifg-1*	Organelle organization and biogenesis	3.75E-03
*sir-2.1*	Lipid transport	2.44E-04
wip-1*	Chromatin assembly or disassembly	1.99E-02
*skn-1*	Chromatin assembly or disassembly	3.56E-04
*vha-6*	Regulation of metabolic process	3.84E-02
*W01B11.3*	Establishment of protein localization	1.93E-04
W06B11.3*	Fatty acid metabolic process	6.78E-03
*rpl-30*	Chromatin assembly or disassembly	3.02E-03
*tag-300*	Cytoskeleton organization and biogenesis	2.59E-02
*Y42G9A.4*	Chromatin assembly or disassembly	3.32E-02
*gdi-1*	Secondary metabolic process	1.98E-02
*spl-1*	Sulfur metabolic process	2.33E-02
*pod-1*	Intracellular protein transport	2.04E-02
*lrs-2*	Intracellular protein transport	2.04E-02
*let-60*	Nucleotide-excision repair	1.11E-02

The first column lists longevity genes, column 2 shows the most highly enriched Gene Ontology biological process in subnetworks containing that gene, and the *P*-value of the enrichment (hypergeometric test with FDR correction) is shown in column 3. Genes with no previously known manual Gene Ontology biological process annotation are indicated with an asterisk.

## Conclusions

Aging results not from individual genes acting in isolation of one another, but from the combined activity of sets of associated genes representing a multiplicity of different biological pathways. For the most part, the organization and function of these aging-related pathways remain poorly understood. In particular, the role of most longevity genes in aging is still unknown.

In this work, we showed that high-throughput information about which genes are likely associated with which other genes - in the form of a functional interaction network - can yield new insights into the transcriptional programs of aging. We identified modular subnetworks associated with worm aging - highly interconnected groups of genes that change activity with age - and showed that they are effective biomarkers for predicting worm age on the basis of gene expression. In particular, they outperform biomarkers of aging based on the activity of single genes or regular subnetworks. Furthermore, we found that modular subnetwork biomarkers were significantly enriched for known longevity genes. Thus, modular subnetwork biomarkers can provide a molecular context for each longevity gene in aging - in effect, each longevity subnetwork constitutes a biological hypothesis as to which genes interact with known longevity genes in some common age-related function.

This work is the first to use a new subnetwork performance criterion that incorporates modularity into the expression for subnetwork score, and the first to integrate network information with gene expression data to identify biomarkers of aging. The subnetwork biomarkers identified by our method are highly conserved across studies, and this opens the door to studying longevity genes - or indeed, any age-related gene set of interest - over a range of different health and disease conditions. In particular, we are interested in investigating the different subnetworks associated with longevity genes in diseases like cancer, and in aging across species.

## Materials and methods

### Code

Code for most simulations was written in Matlab R2008b and is available on the supplementary website [37]. For support vector regression experiments, we used the Matlab wrapper to LIBSVM [42]. We analyzed gene sets for enriched gene ontology using the topGO package (version 1.10.1) [43] in R 2.8.0. Subnetworks were visualized using NAViGaTOR version 2.1.7 [35,44].

### Data sets

#### Microarray experiments

Aging expression datasets for two recent studies were downloaded from the Gene Expression Omnibus [45]. From Golden *et al. *[2], we obtained data for 104 microarrays of individual wild-type (N2) worms over 7 ages (9 to 17 microarrays per age). From Budovskaya *et al. *[21], we obtained 16 microarrays of pooled sterile (fer-15) worms over 4 ages (4 microarrays per age). For both studies, we discarded probesets containing more than 30% missing values for some age group.

#### Interaction network

Functional interactions for *C. elegans *ORFs were downloaded from WormNet [46]. The network used in our analyses consists of the largest connected component of the network formed from all WormNet ORFs represented by some probeset in two separate worm aging microarray studies [2,21], and represents 12,808 distinct *C. elegans *ORFs and 275,525 interactions.

#### Longevity genes

We obtained **L1**, our high confidence set of genes that extend lifespan when perturbed or knocked out, from the recent list compiled in [47]. In total, 233 genetic perturbations that extend lifespan belonged to the largest connected component of WormNet made up of genes covered by both expression studies. We constructed **L2**, our larger set of longevity genes, by taking the union of **L1 **and the set of mutations that affect worm lifespan downloaded from the GenAge database [20]. This yielded 494 genes that either shorten or extend lifespan when perturbed (and are annotated to the network we use). Both gene lists are available in Additional file 4.

### Subnetwork analyses

#### Subnetwork search parameters

##### Seed genes

Previous methods [11,18] seed the subnetwork search process at a random subset of genes on the network; a problem with this approach is that different choices of seed genes might yield substantially different significant subnetworks. To avoid this bias, we grew subnetworks seeded from every node of the interaction network. For all machine learning tests, the total set of significant subnetworks was reduced to a non-redundant set - that is, if two significant subnetworks shared more than 25% overlap (as measured with the Jaccard index), the lower-scoring subnetwork was deleted from the set of candidate features.

##### Stopping criteria

For modular subnetworks grown iteratively out from a seed node, the search was halted when there were no nodes that would increase both subnetwork modularity and class relevance. For regular subnetworks, the search was halted when there were no nodes that would increase the subnetwork score (class relevance) past some threshold r (r = 0.01, 0.02, 0.05, 0.1 and 0.2 for regular subnetworks r1 to r5), or when there were no remaining local nodes (that is, nodes at most two edges away from the seed).

#### Identifying significant subnetworks

We calculate subnetwork significance using both self-contained and competitive gene set tests [8,48]. Our competitive test is identical to that used in [11], and our self-contained test is more stringent - we use the method suggested in [18].

For the self-contained test, we randomized the assignment of ages to worms (samples), and then repeated the search for subnetworks starting from each network node. The subnetwork score of the original subnetwork determined from the true data was then ranked against the corresponding subnetworks determined from the artificial data that seeded from the same gene. This process was repeated 1,000 times.

For the competitive test, we generated 100 artificial interactomes by randomizing the assignment of gene names to nodes on the functional interaction network and recalculating the weight for each network edge based on the new genes that flanked it (only for modular networks - regular networks do not use edge information). We repeated the search for significant subnetworks on each artificial interactome. Scores for subnetworks determined from the true interactome were ranked against the scores of all subnetworks generated from the artificial interactomes.

Subnetworks were considered significant if they achieved *P *< 0.001 on the local self-contained test and *P *< 0.05 on the global competitive test.

#### Machine learning comparisons

We used ε-insensitive SVR algorithms [49] to learn worm age as a function of the activity of regular subnetworks, modular subnetworks or differentially expressed genes. All SVRs were trained using a linear kernel and the default parameters provided by LIBSVM [42]. For SVR features made up of subnetworks, subnetwork activity for a sample was calculated as the mean activity of all the genes in the subnetwork.

#### GO and KEGG enrichment analyses

The union of all genes present in some significant modular subnetwork (*β *= 250; derived using data from [2]) was compared with the background network, that is, the set of 12,808 genes present in the largest connected component of the network formed from all WormNet ORFs represented by some probeset in both microarray studies [2,21].

Because there is a lot of redundancy in the GO tree, we used the 'elim' method [43] to determine the most specific significant biological process categories (that is, those at the deepest level of the tree), and then controlled for multiple testing using an FDR [34] cutoff of 0.05. For KEGG, we calculated an enrichment *P*-value for each term using the hypergeometric test, and again controlled for multiple testing using an FDR cutoff of 0.05.

## Abbreviations

FDR: false discovery rate; GO: Gene Ontology; KEGG: Kyoto Encyclopedia of Genes and Genomes; MSE: mean-squared error; ORF: open reading frame; SCC: squared correlation coefficient; SVR: support vector regression.

## Authors' contributions

KF and IJ conceived and designed the study. KF and MK performed research and analyzed data. KF and IJ wrote the paper. All authors read and approved the final manuscript.

## Supplementary Material

Additional file 1 Box-plots showing Support Vector Regression performance of modular subnetworks, regular subnetworks, and genes trained to predict age using wild-type worm data and tested on fer-15 worm data. Click here for file

Additional file 2 Comparing Support Vector Regression performance of modular and regular subnetworks trained to predict age using wild-type worm data and tested on fer-15 worm data. Click here for file

Additional file 3 Significant modular subnetworks identified using a modularity coefficient of β = 250, grown using the data in Golden et al. Click here for file

Additional file 4 Known *Caenorhabditis elegans *longevity genes. Click here for file

Additional file 5 Putative Gene Ontology (GO) Biological Process (BP) annotations for longevity genes. Click here for file
