# Supplementary material for: Inferring the functions of longevity genes with modular subnetwork biomarkers of Caenorhabditis elegans aging
Source: Genome Biol. 2010 Feb 3;11(2):R13. doi: 10.1186/gb-2010-11-2-r13 (PMC2872873; doi:10.1186/gb-2010-11-2-r13)
Supplement: Additional file 1 — Box-plots showing Support Vector Regression performance of modular subnetworks, regular subnetworks, and genes trained to predict age using wild-type worm data and tested on fer-15 worm data. [file gb-2010-11-2-r13-S1.pdf]

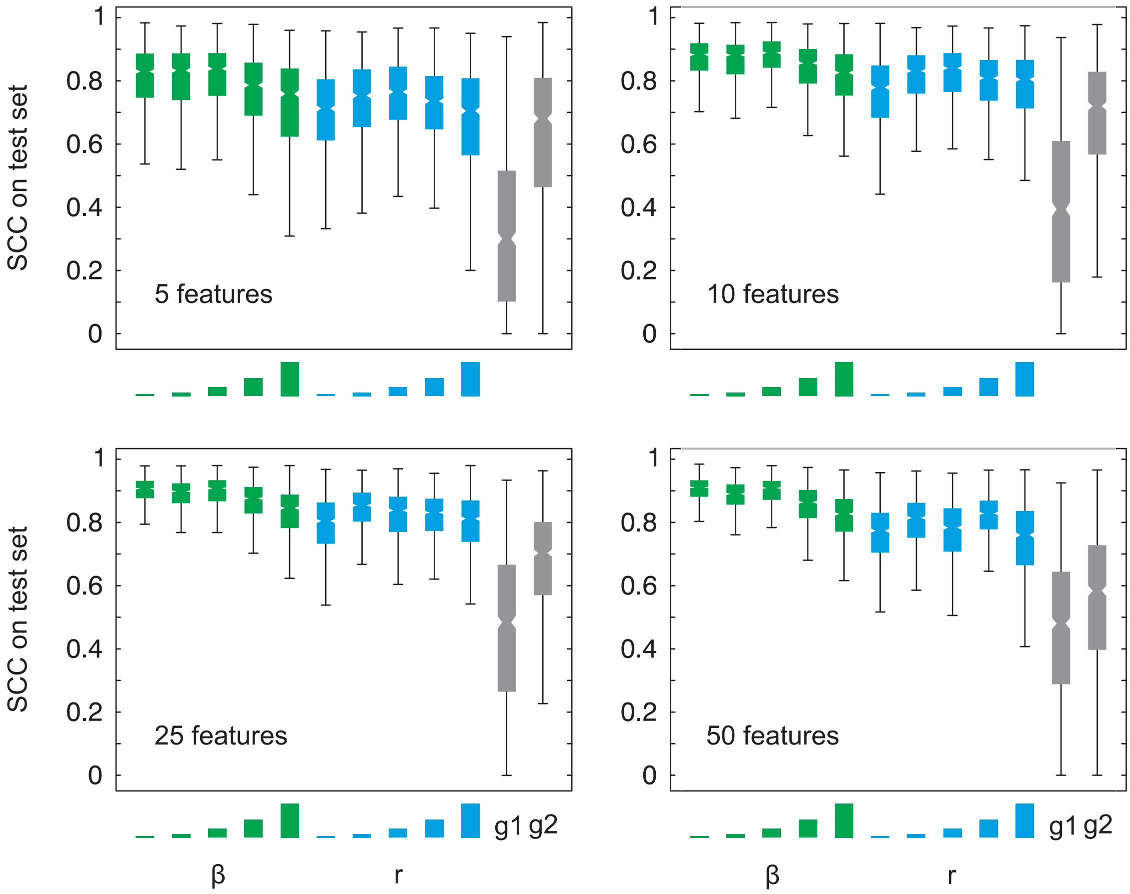

Figure S1 - Boxplots showing Support Vector Regression (SVR) performance of modular subnetworks, regular subnetworks, and genes trained to predict age using wild-type worm data and tested on fer-15 worm data. Modular subnetworks m1-m5 are shown in green, regular subnetworks r1-r5 in blue, and gene sets g1-g2 in gray. Boxplots show the typical performance of 1000 SVR learners for each feature type, quantified using the squared correlation coefficient (SCC) between true and predicted ages. Notches indicate the 95% confidence interval for the medians.
